# Strategies to limit invasive fungal infection in a coronavirus disease 2019 (COVID-19) intensive care unit: The role of infection prevention for renovation and construction in resource-limited settings

**DOI:** 10.1017/ash.2022.35

**Published:** 2022-05-04

**Authors:** Ornnicha Sathitakorn, Surachai Chaononghin, Panipak Katawethiwong, Thanus Pientong, David J. Weber, David K. Warren, Piyaporn Apisarnthanarak, Anucha Apisarnthanarak

**Affiliations:** 1 Division of Infectious Diseases, Faculty of Medicine, Thammasat University, Prathum Thani, Thailand; 2 Gillings School of Global Public Health, University of North Carolina, Chapel Hill, North Carolina, United States; 3 Division of Infectious Diseases, Washington University School of Medicine, St. Louis, Missouri, United States; 4 Division of Diagnostic Radiology, Department of Radiology, Faculty of Medicine Siriraj Hospital, Mahidol University, Bangkok, Thailand

## Abstract

Hospital construction and renovation activities are the main cause of healthcare-associated fungal outbreaks. Infection control risk assessments (ICRAs) for renovation and construction decrease the risk of healthcare-associated fungal outbreaks, but they are typically not performed in developing countries. We reviewed an outbreak investigation to limit the construction-related fungal infections in a COVID-19 ICU in a resource-limited setting.

Invasive pulmonary aspergillosis (CAPA) associated with coronavirus disease 2019 (COVID-19) has been reported to occur in 3.3%–33.3% of intensive care unit (ICU) patients with COVID-19, and CAPA is associated with a 28.5%–100% mortality rate.^1,2^ Immune dysregulation, defective reactive oxygen species production, together with epithelial barrier damage of respiratory tract from COVID-19 immunopathology, are suspected mechanisms predisposing patients to CAPA.^3^ Invasive fungal infection (IFI) is a well-known complication in immunocompromised patients. Major predisposing conditions include corticosteroid therapy, comorbidities (eg, diabetes mellitus, chronic pulmonary disease), and prolonged or inappropriate antibiotic exposure.^4^ Notably, construction and demolition work accounted for 49% of healthcare-associated IFI outbreaks due to fungal spore exposure.^5^ Therefore, the occurrence of 2 or more cases of IFI in a short period (2–6 months) should be considered an outbreak and requires prompt investigation.^6^

## Outbreak investigation

During April 1–30, 2021, at Thammasat University Hospital (Prathum Thani, Thailand), the first case of aspergillosis was detected in a COVID-19 ICU patient, followed by 3 additional cases in the same COVID-19 ICU, accounting for 16% of the 25 patients in the ICU within a month. All 4 patients were diagnosed with probable invasive pulmonary aspergillosis by positive fungal cultures obtained by bronchoalveolar lavage and positive serum galactomannan based on consensus definitions of invasive fungal disease from the European Organization for Research and Treatment of Cancer and the Mycoses Study Group Education and Research Consortium.^7^ The fungal culture of the first patient revealed *Aspergillus flavus*, and cultures from the other 3 patients yielded *Aspergillus fumigatus*. All of these patients died within 2 weeks after positive fungal cultures. After these patients were identified, an outbreak investigation was initiated based on a bundle of key methods for preventing filamentous fungal infection associated with renovation and construction activities.^4^ The hospital epidemiologist performed an infection control risk assessment (ICRA) of renovation and construction activity near the ICU. Initial outbreak investigation steps included air sampling to measure airborne fungal bioburden at the front of ICU, the nursing station, the index patient anterooms, and rooms. Airborne fungal bioburdens from air sampling were 235–290 CFU/m^3^ at all sites. In this hospital, the baseline standard airborne fungal bioburden was <500 CFU/m^3^ for the general unit and <150 CFU/m^3^ for the ICU and operating room.^8^ Air pressures in the COVID-19 unit were checked at the front of ICU, the nursing station, the index patient anterooms, and the index patient rooms and measured 0, 0, −5, and −10 kPa, respectively. The hospital epidemiology team assessed the standard of construction sites using the ICRA for all renovation and construction activities and identified the demolition work including removing the floor covering, ceiling tiles, case work, and new wall construction on the same floor as the COVID-19 ICU. Based on the ICRA matrix, the patient population was classified as the “highest risk group,” and the construction project was classified as type C, leading to a designation of class IV renovation/construction. (The ICRA matrix is available at https://www.ashe.org/icra2.) After inspecting the construction site to identify possible solutions, we instituted appropriate preventive measures.

Following the outbreak investigation, focused preventive efforts to reduce airborne fungal spores using the following interventions: site containment, installation of critical barriers to seal construction areas from clinical areas, cleaning of construction areas, use negative air-pressure handling of the construction site using exhaust fans, and installing portable HEPA filters at the construction site. Pressure at the ICU was recalibrated to be +10 kPa at the nursing station, −10 kPa in all anterooms, and −20 kPa in all patient rooms. Additional interventions included continuous monitoring and serial air sampling for airborne fungal bioburden at the front of ICU, the nursing station, the index patient’s anterooms, and rooms. Our interventions revealed a substantial reduction in airborne fungal bioburden in the air; airborne fungal levels declined to 30–50 CFU/m^3^ at all sites. Continued surveillance for healthcare-associated filamentous fungal infections during renovation and construction was performed for an additional 6 months, and no additional cases of aspergillosis in the same unit occurred.

In conclusion, hospital construction and renovations activities are never ending, and they are the main cause of healthcare-associated fungal outbreaks,^5^ which pose a serious threat to immunocompromised hosts, including high-risk patients (eg, COVID-19 pneumonia patients). Following ICRA for renovation and construction decreases the risk of healthcare-associated fungal outbreaks. However, ICRAs are not typically performed in developing countries, which may lead to healthcare-associated fungal outbreaks in settings such as ICUs, hematologic wards, and renal transplant units.^6^ Although uncommon, healthcare-associated fungal outbreaks must be suspected when 2 or more cases occur in a short period.^6^ Early outbreak investigation and identification of the source are likely to play a key role in limiting the spread of healthcare-associated fungal outbreaks. Such strategies, together with a full compliance with recommendations from a multidisciplinary coordination team, will help limit the construction-related fungal infection in a COVID-19 ICU. National strategies are needed in developing countries to promote the use of ICRA for renovation and construction to limit healthcare-associated fungal outbreaks.
